# Management of Severe Rectal Variceal Bleeding in a Patient With Alcohol-Related Cirrhosis: A Case Report

**DOI:** 10.7759/cureus.84339

**Published:** 2025-05-18

**Authors:** Urmimala Chaudhuri, Diane S Casini, Sean-Patrick A Prince, Sangeeta Agrawal

**Affiliations:** 1 Internal Medicine Residency Program, Wright State University, Dayton, USA; 2 Internal Medicine, Wright State University/Premier Health, Dayton, USA; 3 Gastroenterology, Wright State University Boonshoft School of Medicine, Dayton, USA; 4 Internal Medicine-Gastroenterology, Dayton Veterans Affairs (VA) Medical Center, Wright State University, Dayton, USA

**Keywords:** gastrointestinal bleeding, live cirrhosis, portal hypertension, rectal varices, transjugular intrahepatic portosystemic shunt (tips)

## Abstract

Rectal varices (RV) are portosystemic collaterals that are a result of portal hypertension. RV prevalence has been reported between 63% and 94% among patients with cirrhosis; however, clinically significant bleeding is a rare complication and occurs in about 0.5% to 5% of patients. To date, no specific evidence-based guidelines exist for the management of bleeding RV, which can be life-threatening with a high morbidity and mortality. Current management involves a multidisciplinary team and urgent endoscopic evaluation in all patients. Here, we present a rare case of severe RV bleeding in a patient with cirrhosis presumed secondary to alcohol use, who ultimately expired despite multiple endoscopic interventions and salvage therapies. The patient’s deteriorating condition from severe hemorrhagic shock and limitation of procedures given poor candidacy for transjugular intrahepatic portosystemic shunt (TIPS) highlights the limited treatment options available in such advanced cases. It warrants further discussions on establishing dedicated guidelines and advancing therapies for refractory cases.

## Introduction

Rectal varices (RV) are one manifestation of portosystemic collateral veins that form in response to portal hypertension. These dilated submucosal veins develop at sites of communication between the portal and systemic venous systems, including the rectum, esophagus, and stomach [1]. As portal venous pressures exceed 12 mmHg, blood is diverted through collateral pathways, including rectal venous anastomosis, to reduce resistance [1]. RV have been reported in 38% to 56% of patients with cirrhosis, though clinically significant bleeding remains rare and occurs in about 0.5% to 5% of patients [2]. When bleeding does occur, it can be life-threatening due to the combination of high pressure, thin vessel walls, and potential for mucosal ulceration. Despite their clinical significance, there are no established clinical guidelines for management strategies [3]. While esophageal and gastric varices are the more common source of gastrointestinal bleeding in cirrhosis, RV pose unique diagnostic and therapeutic challenges, especially when resistant to conventional therapies. Our case report highlights the diagnostic and therapeutic complexities in managing a patient with alcohol-related cirrhosis and severe rectal variceal bleeding.

## Case presentation

**Figure 1:**
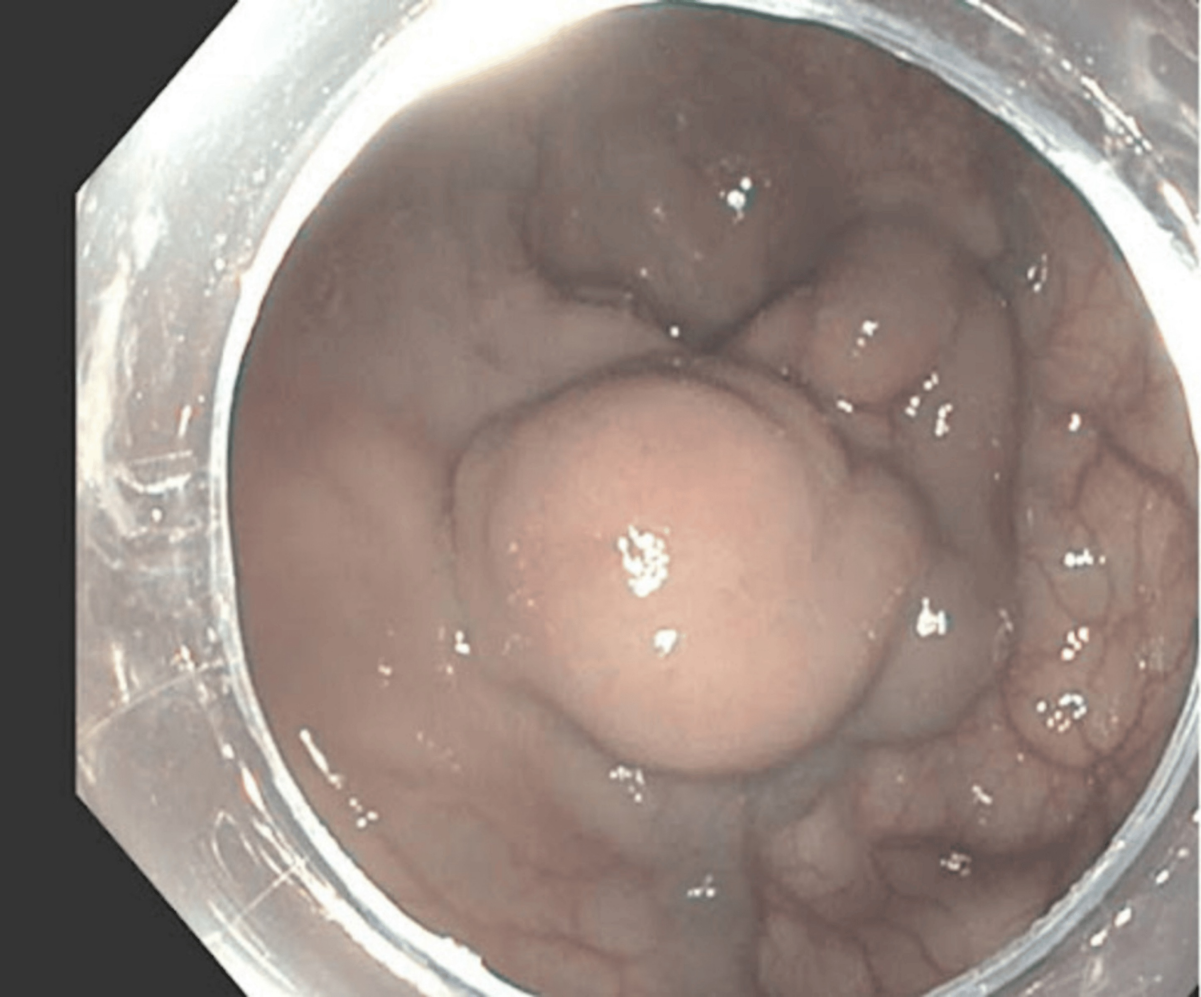
Endoscopic image of a large rectal varix visualized on sigmoidoscopy with an overlying ulceration. This lesion was identified as the presumed source of bleeding and prompted endoscopic intervention with clipping and initiation of octreotide infusion.

**Figure 2:**
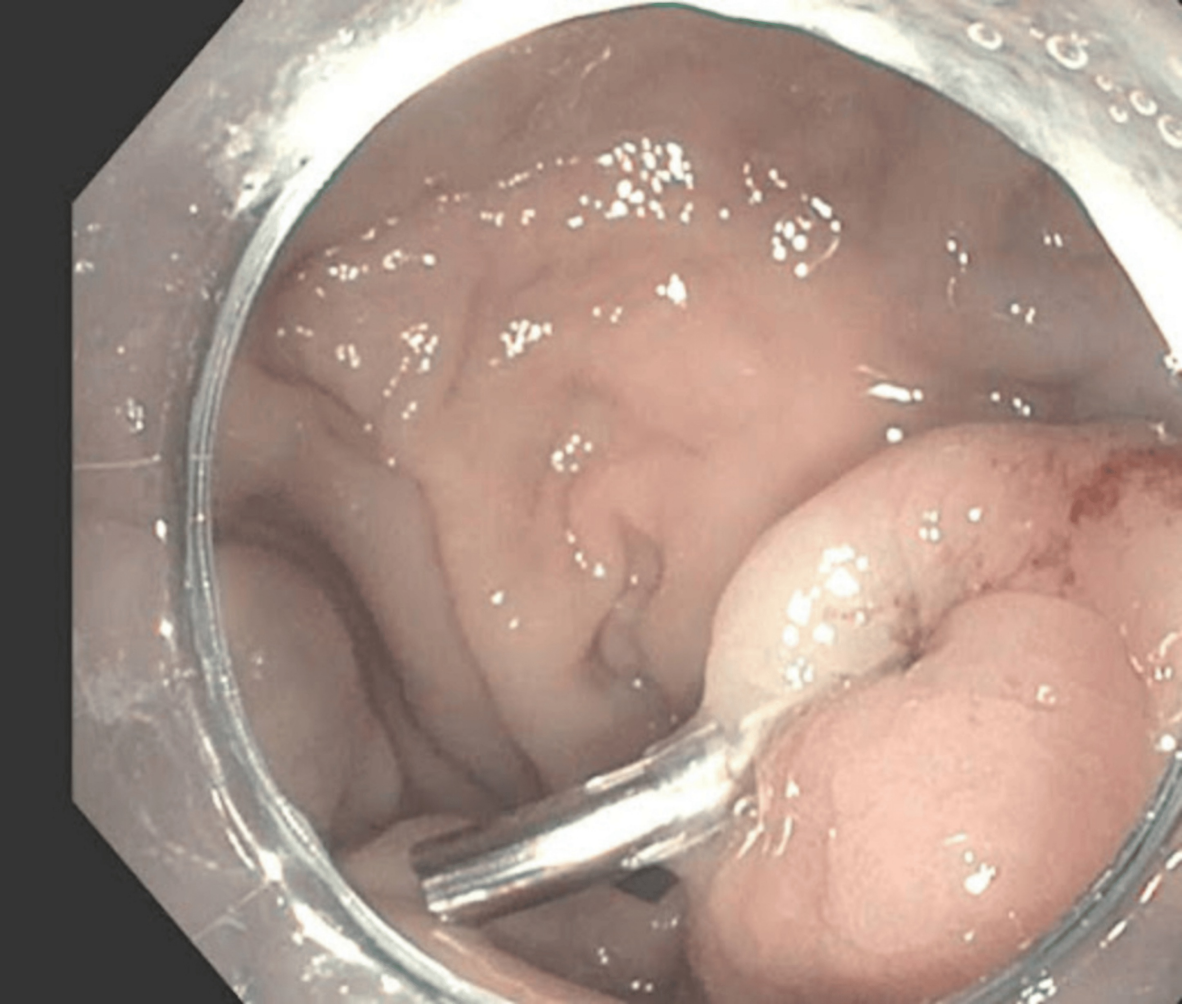
Endoscopic image showing deployment of an endoscopic clip on the ulcerated rectal varix. Despite clipping and medical therapy, the patient continued to have massive rectal bleeding, indicating refractory hemorrhage.

A 52-year-old male patient with liver cirrhosis, presumed secondary to alcohol use, presented with a two-week history of abdominal discomfort and maroon-colored stools. He reported consuming 12 beers daily prior to hospitalization. His Model for End-Stage Liver Disease-Sodium (MELD-Na) score was 19. On admission, he was hemodynamically stable except for mild tachycardia. On physical exam, the patient was alert and oriented. His abdomen was soft, non-tender, and nondistended without organomegaly. Laboratory workup revealed normocytic anemia (6.9 g/dL), thrombocytopenia (54 x 10^9/L), mild hyponatremia (131 mmol/L), total bilirubin of 2.7 mg/dL, aspartate aminotransferase (AST) of 148 U/L, and normal alanine aminotransferase (ALT). Laboratory workup also revealed coagulopathy with prothrombin time international normalized ratio (INR) of 1.7 and an ethanol level of 238 (Table 1). Computed tomography (CT) imaging revealed irregular liver edges suspicious for hepatic cirrhosis with a right lobe mass measuring 3.9 cm and signs of portal hypertension, including a small amount of perihepatic, perisplenic, and pelvic ascites with prominent venous RV without splenomegaly. The patient underwent an esophagogastroduodenoscopy (EGD) and colonoscopy, revealing non-bleeding esophageal varices, mild portal hypertensive gastropathy, and a large rectal varix with an overlying ulcer. Figure 1 shows the visualized rectal varix during sigmoidoscopy, which was the suspected source of bleeding. The ulcer over the varix was clipped as shown in Figure 2. Post procedure, the patient was started on a continuous octreotide infusion for 72 hours. 

**Table 1:** The patient's baseline lab results on admission

Laboratory	Result	Reference range
White blood cell (WBC) count	5.5 K/uL	3.5-10.9 K/uL
Red blood cell (RBC)	2.06 M/uL	4.14-5.8 M/uL
Hemoglobin	6.9 g/dL	13-17.7 g/dL
Hematocrit	20.10%	37.5-51%
Platelet count	54 K/uL	140-400 K/uL
Sodium	131 mEq/L	135-148 mEq/L
Potassium	3.7 mEq/L	3.4-5.3 mEq/L
Chloride	100 mEq/L	96-110 mEq/L
Carbon dioxide	18 mEq/L	19-32 mEq/L
Blood urea nitrogen (BUN)	12 mg/dL	3-29 mg/dL
Creatinine	0.6 mg/dL	0.5-1.4 mg/dL
Glucose	124 mg/dL	70-99 mg/dL
Calcium	7 mg/dL	8.5-10.5 mg/dL
Anion gap	13	5-15
Total bilirubin	2.7 mg/dL	0.0-1.2 mg/dL
Total protein	5.7 g/dL	6-8.3 g/dL
Albumin	2.6 g/dL	3.5-5.2 g/dL
Aspartate aminotransferase (*AST*)	148 U/L	0-46 U/L
Alanine aminotransferase (ALT)	25 U/L	0-60 U/L
Alkaline phosphatase	115 U/L	23-144 U/L
International normalized ratio (INR)	1.7	0.9-1.1
Ethanol	238 mg/dL	<10 mg/dL

To our knowledge, the patient did not have a prior history of chronic constipation or hemorrhoids. It was felt that the RV were caused by portal hypertension in the setting of liver cirrhosis. Serologic testing for underlying liver disease, including viral hepatitis B and C and autoimmune markers, was not performed due to the emergent nature of the presentation, the absence of relevant risk factors, and the patient’s rapid clinical decline. 

**Figure 3:**
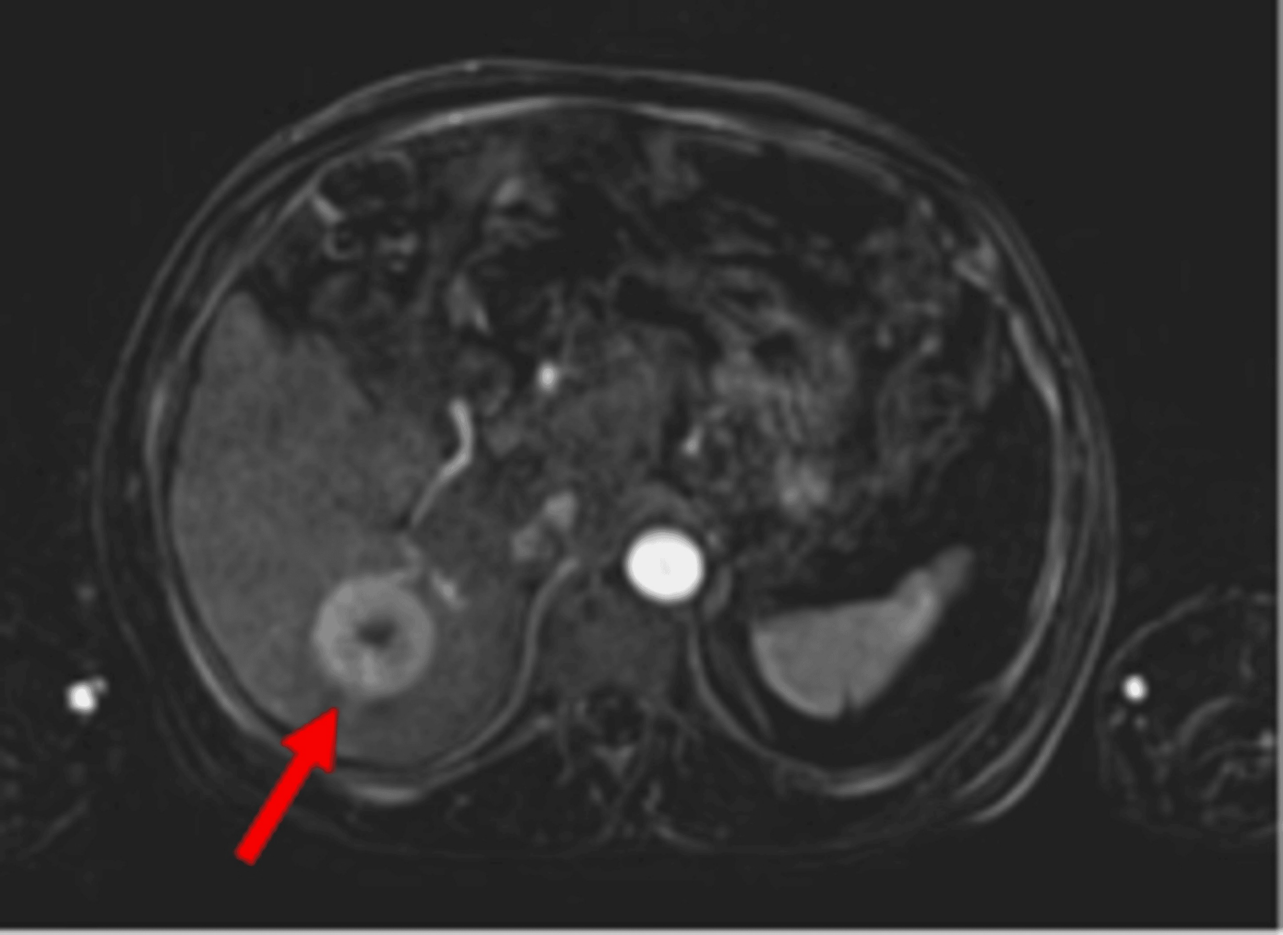
Axial MRI of the liver showing a 4.1 x 3.9 cm arterial enhancing lesion with delayed washout in the right hepatic lobe (red arrow), consistent with hepatocellular carcinoma. The presence of HCC influenced the decision-making regarding TIPS candidacy, given its association with poor prognosis and increased procedural risk. HCC: hepatocellular carcinoma; TIPS: transjugular intrahepatic portosystemic shunt

Magnetic resonance imaging (MRI) of the abdomen with and without contrast was obtained to further evaluate the hepatic lesions. The patient was found to have a T2 isointense and T1 hyperintense lesion in the right hepatic lobe measuring 4.1 x 3.9 cm (Figure 3) with arterial hyperenhancement. He also had a smaller lesion in the left hepatic lobe measuring 2.5 x 2.2 cm with arterial hyperenhancement and delayed washout. Per radiology, the lesions were highly suspicious for hepatocellular carcinoma. The MRI also noted cirrhosis, hepatic steatosis, and a moderate amount of ascites. 

Two days into hospitalization, the patient was transferred to the intensive care unit (ICU) for alcohol withdrawal-related seizures and subsequently decompensated due to hemorrhagic shock from large-volume rectal bleeding. He required 10 units of packed red blood cells, two units of fresh frozen plasma (FFP), one unit of platelets, and one unit of cryoprecipitate. Following failed endoscopic intervention, gastroenterology recommended emergent transjugular intrahepatic portosystemic shunt (TIPS) and interventional radiology (IR)-guided embolization. However, TIPS was initially deferred due to the patient’s elevated ammonia level of 173 umol/L, raising concern for precipitating or worsening hepatic encephalopathy. Additionally, MRI findings (Figure 3) revealed the right hepatic lobe lesion was concerning for hepatocellular carcinoma (HCC), which further complicated his candidacy for TIPS due to poor prognosis. The patient was intubated, with absent response to noxious stimuli. While his cough and gag reflexes were preserved, his corneal reflex was absent, suggesting evolving neurologic decline. 

The patient soon developed another episode of massive bleeding per rectum, prompting activation of the massive transfusion protocol. He received 23 units of packed red blood cells, 12 units of FFP, two units of platelets, and two units of cryoprecipitate in response to ongoing hemodynamic instability, characterized by systolic blood pressures persistently below 90 mmHg and rising lactate levels peaking at 5.9 mmol/L. Gastroenterology was urgently called to the bedside, and repeat flexible sigmoidoscopy again demonstrated a large rectal varix. Attempts at endoscopic hemostasis were unsuccessful. The surgical team was emergently consulted and attempted direct suturing of the rectal varix, which slowed but did not stop the bleeding. Subsequently, 6 mL of sodium tetradecyl sclerosing agent was injected, but this too failed to achieve hemostasis. The patient’s hemodynamics continued to decline, and as a last resort, a Blakemore tube was inserted into the rectum under direct visualization, which temporarily stabilized the patient’s hemodynamics and provided short-term bleeding control. 

Despite the patient’s poor candidacy, TIPS was pursued as salvage therapy after failed endoscopic and surgical attempts for bleeding control. Unfortunately, the patient began oozing blood from multiple access sites during the procedure, requiring additional transfusions. The procedure was aborted, and the patient expired shortly after due to uncontrollable hemorrhagic shock. 

## Discussion

This case highlights the challenges in managing life-threatening rectal variceal bleeding in cirrhotic patients. Although RV are relatively common in cirrhosis (up to 56%), clinically significant bleeding occurs in fewer than 5% of cases [2]. When bleeding does occur, it can be catastrophic. 

There are no dedicated guidelines specific to the management of rectal variceal bleeding [3]. As a result, the management of RV is often surmised from the general management principles of variceal bleeding in portal hypertension, specifically in the context of esophageal or gastric varices [2]. These principles include a multidisciplinary approach involving gastroenterologists, interventional radiologists, and surgeons; urgent hemodynamic resuscitation; early endoscopic intervention; and prevention and treatment of complications [3, 4]. 

Endoscopic injection sclerotherapy (EIS) is often favored over band ligation for RV bleeding, although there is no consensus on the optimal sclerosant concentration or volume [5]. Band ligation remains an alternative, but it carries the risk of deep ulceration or incomplete obliteration [6]. While TIPS is the standard of care for esophageal variceal bleeding, its role in managing RV is less clearly defined and largely limited to case reports. TIPS remains the most effective option for portal decompression and achieving homeostasis [7]. When feasible, adjunctive embolization may be considered to further reduce the risk of rebleeding [7, 8]. However, TIPS carries risks, particularly in patients with high MELD scores or suspected HCC, as was the case in our patient. Prior studies have shown limited benefit of TIPS in patients with MELD scores >18 and concurrent HCC, consistent with this patient’s outcome. Surgical options such as underrunning sutures may be considered in selected patients with preserved hepatic reserve; however, these carry an increased operative risk [9]. Pharmacologic prophylaxis with non-selective beta blockers is commonly used, though there are no evidence-based recommendations specific to RV. 

In the case presented above, TIPS was attempted as salvage therapy to control refractory bleeding despite the patient’s poor candidacy due to a high MELD-Na score and hepatic lesions suspicious for cirrhosis. The patient’s deteriorating condition and complications from the procedure highlight the limited treatment options available in such advanced cases. Furthermore, it warrants discussions on establishing dedicated guidelines and advancing therapies for severe and refractory cases. 

## Conclusions

Severe rectal variceal bleeding is a rare but devastating complication of portal hypertension in cirrhotic patients. Our case highlights the limited treatment options available in such advanced cases, particularly in patients with advanced liver disease and poor TIPS candidacy, and resultant poor outcomes that reflect these limitations. The importance of a multidisciplinary approach and the need for early, aggressive management in high-risk cirrhotic patients to optimize outcomes are critical, though prognosis remains poor in severe cases. Our case further emphasizes the need for dedicated management guidelines and research into optimal therapeutic strategies for RV bleeding. Continuing development of dedicated devices such as endoscopic ultrasound (EUS)-guided coils or tools for rectal variceal hemostasis may be useful in future practice. Additionally, the creation of risk stratification tools is needed to guide early intervention decisions.
